# The Importance of Biotinylation for the Suitability of Cationic and Neutral Fourth-Generation Polyamidoamine Dendrimers as Targeted Drug Carriers in the Therapy of Glioma and Liver Cancer

**DOI:** 10.3390/molecules29184293

**Published:** 2024-09-10

**Authors:** Łukasz Uram, Magdalena Twardowska, Żaneta Szymaszek, Maria Misiorek, Andrzej Łyskowski, Zuzanna Setkowicz, Zuzanna Rauk, Stanisław Wołowiec

**Affiliations:** 1 The Faculty of Chemistry, Rzeszow University of Technology, Powstańców Warszawy 6 Ave., 35-959 Rzeszow, Poland; luram@prz.edu.pl (Ł.U.); magdalenatwardowska5@gmail.com (M.T.); zaneta.szymaszek212@gmail.com (Ż.S.); mczygier@prz.edu.pl (M.M.); alyskowski@prz.edu.pl (A.Ł.); 2 Institute of Zoology and Biomedical Research, Jagiellonian University, 30-387 Krakow, Poland; zuzanna.setkowicz@uj.edu.pl (Z.S.); zuzanna.rauk@doctoral.uj.edu.pl (Z.R.); 3 Medical College, University of Rzeszow, 1a Warzywna Street, 35-310 Rzeszow, Poland

**Keywords:** PAMAM dendrimers, biotinylation, SMVT, *Caenorhabditis elegans*, glioma U-118 MG, hepatocarcinoma HepG2, HaCaT keratinocytes, cytotoxicity, cellular uptake, TMZ-resistant glioma

## Abstract

In this study, we hypothesized that biotinylated and/or glycidol-flanked fourth-generation polyamidoamine (PAMAM G4) dendrimers could be a tool for efficient drug transport into glioma and liver cancer cells. For this purpose, native PAMAM (G4) dendrimers, biotinylated (G4B), glycidylated (G4gl), and biotinylated and glycidylated (G4Bgl), were synthesized, and their cytotoxicity, uptake, and accumulation in vitro and in vivo were studied in relation to the transport mediated by the sodium-dependent multivitamin transporter (SMVT). The studies showed that the human temozolomide-resistant glioma cell line (U-118 MG) and hepatocellular carcinoma cell line (HepG2) indicated a higher amount of SMVT than human HaCaT keratinocytes (HaCaTs) used as a model of normal cells. The G4gl and G4Bgl dendrimers were highly biocompatible in vitro (they did not affect proliferation and mitochondrial activity) against HaCaT and U-118 MG glioma cells and in vivo (against *Caenorhabditis elegans* and Wistar rats). The studied compounds penetrated efficiently into all studied cell lines, but inconsistently with the uptake pattern observed for biotin and disproportionately for the level of SMVT. G4Bgl was taken up and accumulated after 48 h to the highest degree in glioma U-118 MG cells, where it was distributed in the whole cell area, including the nuclei. It did not induce resistance symptoms in glioma cells, unlike HepG2 cells. Based on studies on Wistar rats, there are indications that it can also penetrate the blood–brain barrier and act in the central nervous system area. Therefore, it might be a promising candidate for a carrier of therapeutic agents in glioma therapy. In turn, visualization with a confocal microscope showed that biotinylated G4B penetrated efficiently into the body of *C. elegans,* and it may be a useful vehicle for drugs used in anthelmintic therapy.

## 1. Introduction

Cancer is one of the most significant global health challenges and is a leading cause of death worldwide, accounting for one in six deaths globally (16.8%) [1,2]. Predictions indicate that by 2040 the number of new cancer cases is likely to exceed 30 million [3]. Glioblastoma multiforme (GBM), identified as a grade IV astrocytoma, is the predominant, aggressive, and fatal primary brain tumor among adults [4]. Despite significant progress in surgical techniques and radio- and chemotherapy over the last decade, the prognosis for patients with GBM remains bleak, with a median survival of only 12–15 months [5] and a 5-year patient survival rate of <3% [6]. The invasive nature of the surgical intervention and the lack of clear boundaries surrounding the tumor make it challenging to achieve a complete removal. Furthermore, the postoperative recurrence rate exceeds 90% [7]. The accepted standard treatment regimen for newly diagnosed GBM patients involves surgical resection, followed by radiotherapy and adjuvant chemotherapy with the alkylating agent temozolomide (TMZ) [8]. The management of GBM is particularly challenging due to the presence of the blood–brain barrier (BBB)/blood–brain tumor barrier (BBTB) and the complex GBM microenvironment. These factors create significant obstacles to the effective delivery of anticancer drugs to the tumor, severely restricting the range of available treatment options [9]. 

The liver is the sixth most common location of cancer in humans and the third leading cause of cancer-related mortality [10]. Hepatocellular carcinoma (HCC), cholangiocarcinoma, and metastatic liver cancer are the three main types of liver cancer (LC) [11]. Among them, HCC is responsible for nearly 90% of cases [12]. The available systemic treatment options, such as surgery, chemotherapy, radiofrequency ablation, and immunotherapy, have shown insufficient effectiveness in treating patients suffering from LC [13]. Early diagnosis is linked with more than 70% survival rates at 5 years, whereas advanced stages may have less than 20% survival rates over the same period [14]. Approximately 70% of patients experience recurrences and metastases following surgical resection [15]. Traditional first-line chemotherapy for HCC typically includes fluorouracil, folinic acid, and oxaliplatin, but these therapies have not delivered satisfactory outcomes [10]. For the past decade, sorafenib has been the primary treatment option. During this time, the median overall survival of sorafenib monotherapy has increased from 10.7 months to 14.7 months [15]. An important factor leading to the poor prognosis of HCC is drug resistance [16].

Many standard drugs show relatively low efficacy in cancer therapy due to their unfavorable properties, and the induced resistance to the drugs. These features include low water solubility, poor pharmacokinetics, many side effects, and systemic toxicity. Chemotherapy is usually non-selective, thus leading to systemic toxicity. Although many drugs are approved after in vivo studies, clinical trials reveal too many side effects. In consequence, the medicine cannot be placed on the market, as in the case of bevacizumab [17]. The use of small molecules, including genes, small RNAs, and plasmids is also finding poor satisfaction, due to insufficient in vivo stability [18]. Another problem is the distribution of these drugs in the tumor area [19]. Tumors often show resistance to most drugs, known as multidrug resistance. It is usually the result of multiple mechanisms related to affected drug uptake, drug metabolism, and excessive exocytosis. Associated with these processes are, among other things, ABC proteins or lysosomes, in the case of hydrophobic drugs, causing increased efflux of the drug from the tumor. Among the ABC proteins, one of the main concerns is P-glycoprotein, present in many types of tumors [20,21].

Therefore, to improve the properties and increase the efficacy of anticancer drugs, they may be transported by special carriers. Particles that fulfill the criteria for their use as drug delivery systems are poly(amidoamine) (PAMAM) dendrimers. Compared to traditional, linear polymers, they exhibit significantly better physical and chemical properties. In addition to being monodisperse, they are also characterized by a homogeneous and well-defined size and shape. Their nanoscale size is similar to many biological structures, which facilitates their penetration into the cells and limits exocytosis as well as the crossing of the BBB [22,23]. Moreover, dendrimers can enhance the effect of increased permeability and retention, which further supports their penetration into cancer cells. The dendrimer structure also has useful properties, such as empty spaces between the dendrons or the presence of surface functional groups to which drugs can be attached [24]. The functional groups can be modified to improve the water solubility, biocompatibility, and reactivity of the dendrimers [25]. Many studies have shown that dendrimers enhance the toxicity of anticancer drugs and tumor penetration, and decrease undesirable side effects [26].

Although PAMAM dendrimers show great promise as drug delivery systems, they also indicate generation-dependent cytotoxicity, connected with positive surface charge and concentration [27,28]. Higher-generation, cationic dendrimers with positive surface charges exhibited greater cytotoxicity, unlike those with neutral or anionic charges, such as carboxylic acid or hydroxyl-terminated polyamidoamines [29,30]. The positively charged cationic dendrimers strongly interact with anionic lipid membranes, leading to nanopore formation, an increase in cellular reactive oxygen species (ROS), and finally apoptosis [31,32]. To reduce this cytotoxicity, numerous modifications to the chemical structure of dendrimers have been proposed. Several studies have demonstrated that the cytotoxicity of dendrimers was significantly reduced by PAMAM surface modifications with molecules such as PEG [33], folic acid [34], hyaluronic acid [35], or saccharides [36]. It can be inferred that the attachment of compounds containing hydroxyl (OH) or carboxyl (COOH) groups on their surface allows for an increase in dendrimer biocompatibility. Furthermore, fourth-generation PAMAM dendrimers terminated with hydroxyl groups (G4-OH) have been demonstrated to exhibit efficient permeation through tissues, excretion in an intact form by the kidney, and localization to actively phagocytic macrophages and microglia in a multitude of animal models [37,38,39].

Additionally, small molecule targeted therapies are a fundamental aspect of modern medicine, offering effective treatments for various diseases [39]. This alternative strategy can also be applied to combat cancer cells, targeting their increased metabolic activity and rapid growth, which involve the uptake and internalization of vitamins through overexpressed receptors on specific cells [40]. Biotin [41], folic acid [42], vitamin B12 [43], and riboflavin [44] are among the vitamins essential for cell division, particularly in tumor cells, and have been identified as potential targeting agents [45]. Biotin, known as vitamin B7 or H, enters cells via different transporters [46]. It is currently believed that the principal transporter for biotin is the sodium-dependent multivitamin transporter (SMVT), which is encoded by the SLC5A6 gene, located on chromosome 2p23.3. The SMVT, a transmembrane protein, facilitates the transport of biotin, pantothenic acid (vitamin B5), and α-lipoic acid across the cell membrane. Its expression is typically higher in many lung, liver, kidney, colon, intestinal, breast and ovarian cancer cells [47]. Biotin can also be transported via an alternative pathway involving the monocarboxylate transporter (MCT-1 or MCT-8), located in both plasma and mitochondrial membranes [48]. SMVT utilizes a sodium-dependent co-transport mechanism to facilitate the uptake of biotin into cells [41]. Furthermore, biotin might enhance the transport of dendrimers across the blood–brain barrier (BBB) via carrier-mediated endocytosis [49]. Studies revealed that the conjugate dendrimer–biotin was taken up more effectively by HeLa cells compared to the conjugate without biotin [50]. An enhanced effect was also observed in different cell lines treated with biotin-targeted nanoparticles and drugs, A549 [51], L1210FR [52], and in a co-culture model of the BBB [49,53]. A number of studies have demonstrated that the attachment of biotin to various dendrimer–drug constructs can improve their delivery. This is achieved by increasing the uptake of therapeutic molecules by cells, which is facilitated by the active recognition of the transporter [54].

The aim of this study was to investigate the influence of biotin as a targeting molecule on the penetration and accumulation of positively charged or neutral PAMAM G4 dendrimers. Biotinylated PAMAM G4 dendrimers and/or dendrimers furnished with glycidol residues were considered potential carriers of drugs and biologically active agents in the therapy of brain and liver tumors, with glioma and hepatocellular carcinoma.

## 2. Results and Discussion

Polyamidoamine dendrimers (PAMAMs) have been widely tested as drug carriers since 1985 [55]. The ethylenediamine-cored PAMAM dendrimer of the fourth generation (G4) is a suitable carrier for anticancer drugs [56,57]. It has been demonstrated that the primary terminal amine groups of PAMAM dendrimers are responsible for the toxicity of these macromolecules [58]. The eradication of primary amine groups leads to a considerable decrease in PAMAM dendrimer toxicity. This can be achieved, for instance, by the exhaustive glycidylation of amine groups [57]. Previously, we applied the glycidylation of G4 to obtain conjugates of G4 with the anticancer drugs lapatinib, fulvestrant, and paclitaxel to test the anticancer activity of the conjugates [57]. We used optically pure *R*-glycidol instead of racemic or *S*-isomer due to a previously noticed four-fold higher affinity to the cell membrane of human keratinocytes (HaCaTs) and squamous carcinoma cells (SCC-15s). The *R*-glycidylated G3 was highly biocompatible, with no toxicity up to 300 µM concentrations, in contrast to the amine-terminated PAMAM analogs [59]. 

### 2.1. Chemistry

**Figure 1:**
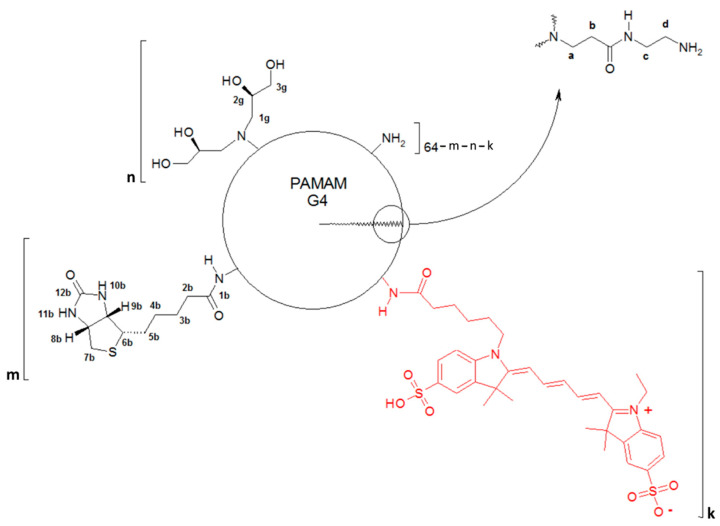
Simplified formula of obtained conjugates: G4 (m, n = 0, k = 1), G4gl (m = 0, n = 63, k = 1), G4B (m, k = 1, n = 0), and G4Bgl (m, k = 1, n = 62). Atom numbering useful for the ^1^H NMR spectrum assignment is given for substituents as well as for the core PAMAM dendrimer subunit (in upper right corner).

PAMAM G4 modified by exhaustive *R*-glycidylation was obtained before and characterized by 1H NMR spectroscopy and Dynamic Light Scattering [57]. The G4gl dendrimer with 128 N-attached 2,3-dihydroxypropyl substituents is a well-water-soluble macromolecule with a 5 nm hydrodynamic diameter and a slightly positive zeta potential, 4 mV. Here, the modified G4 was tested for biological activity. For this purpose, G4 was synthesized and fluorescent-labeled with sulfocyanin5 (20% labeling) and further converted into *R*-glycidylated derivative G4gl and separately into single biotin-attached conjugate G4B. Finally, the G4B was *R*-glycidylated to obtain G4Bgl (Figure 1). 

**Figure 2:**
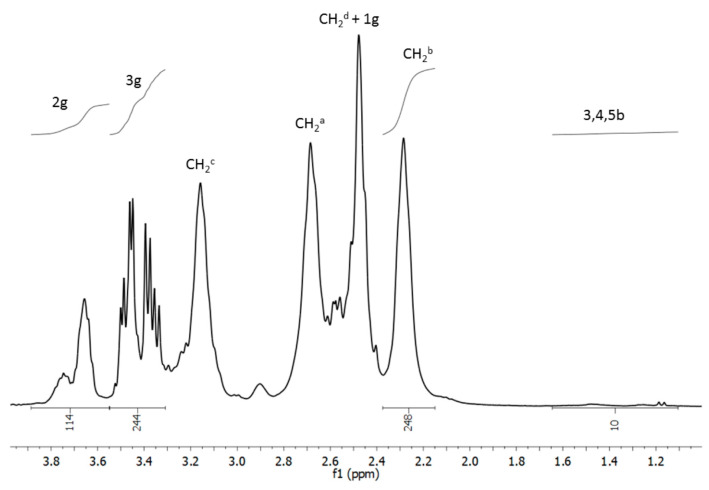
The relevant fragment of the ^1^H NMR spectrum of G4Bgl conjugate in D_2_O. The relevant resonances from biotin (b), R-glycidol-derived 2,3-dihydroxypropyl substituent (g), and the PAMAM G4 core are labeled according to the atom numbering from Figure 1.

The conjugates were characterized by 1H NMR spectroscopy. The PAMAM G4 core CH2b resonance centered at 2.25 ppm was used as an internal integral intensity reference corresponding to 248H. Additionally, the intensities of *R*-glycidol-derived substituent resonances 2g and 3g enabled us to conclude that all remaining primary amine groups of G4B* were almost fully double-substituted with 2,3-dihydroxypropyl residues (integral intensity of 3g resonances was 244H vs. the expected 248H). Additionally, the integral intensity of 3,4,5-H biotin resonances showed the average 1 biotin molecule covalently attached to G4 via an amide bond (Figure 2). All assignments are in accordance with those previously obtained for biotinylated PAMAM G2 and G3 dendrimers [6] and for glycidylated G4 dendrimers [56,57].

### 2.2. SMVT Expression

**Figure 3:**
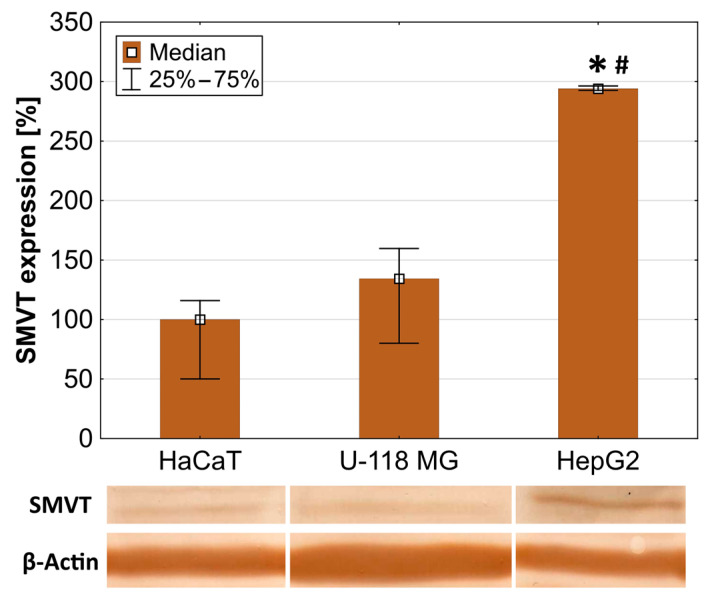
Constitutive expression of SMVT in HaCaT, U-118 MG, and HepG2 cells assessed by Western blot technique. Levels of SMVT were quantified as the percentage of the SMVT level in HaCaT cells. White squares indicate medians; the lower (25%) and upper (75%) quartile ranges are presented as whiskers. Kruskal–Wallis test against HaCaT (* *p* < 0.05) and against U-118 MG cells (# *p* < 0.05).

In order to select an appropriate research model (cell lines indicating increased level of main biotin transporter: sodium-dependent multivitamin transporter—SMVT), Western blot analysis was performed. The obtained data showed that immortalized human keratinocytes (model of normal cells) indicated the lowest level of SMVT. The amount of SMVT was about 40% higher in human, TMZ-resistant glioma U-118 MG cells and nearly three times higher in human liver cancer HepG2 cells (Figure 3).

The obtained results are in agreement with data provided by others. The SMVT expression was proved in HepG2 [60] and HaCaT cells [61]. Grafe et al. showed that HaCaT cells express constitutively the SMVT and indicated that the biotin transport observed in these cells was not restricted to the cell line but is also present in native non-transformed human keratinocytes in primary culture [61]. Human keratinocytes are rapidly dividing cells (doubling time for HaCaTs = 24 h). Because cell proliferation requires a substantial increase in the replication and transcription of DNA, an increased requirement of biotin uptake by proliferating cells for histone biotinylation is necessary [62]. There are no data concerning the expression of SMVT in U-118 MG cells; however, it has been shown that in the U-251 glioma cell line (similar to U-118 MG), SMVT RNA was detected. A remarkably similar quantitative pattern of SMVT RNA (SLC5A6 gene) was presented in the human protein atlas (HaCaT < U-251 MG < HepG2) [63]. Therefore, the described cell lines are appropriate experimental models to study the mechanisms of uptake of biotinylated nanoparticles by normal and cancer cells with varying levels of SMVT.

### 2.3. Dendrimers Cytotoxicity

**Figure 4:**
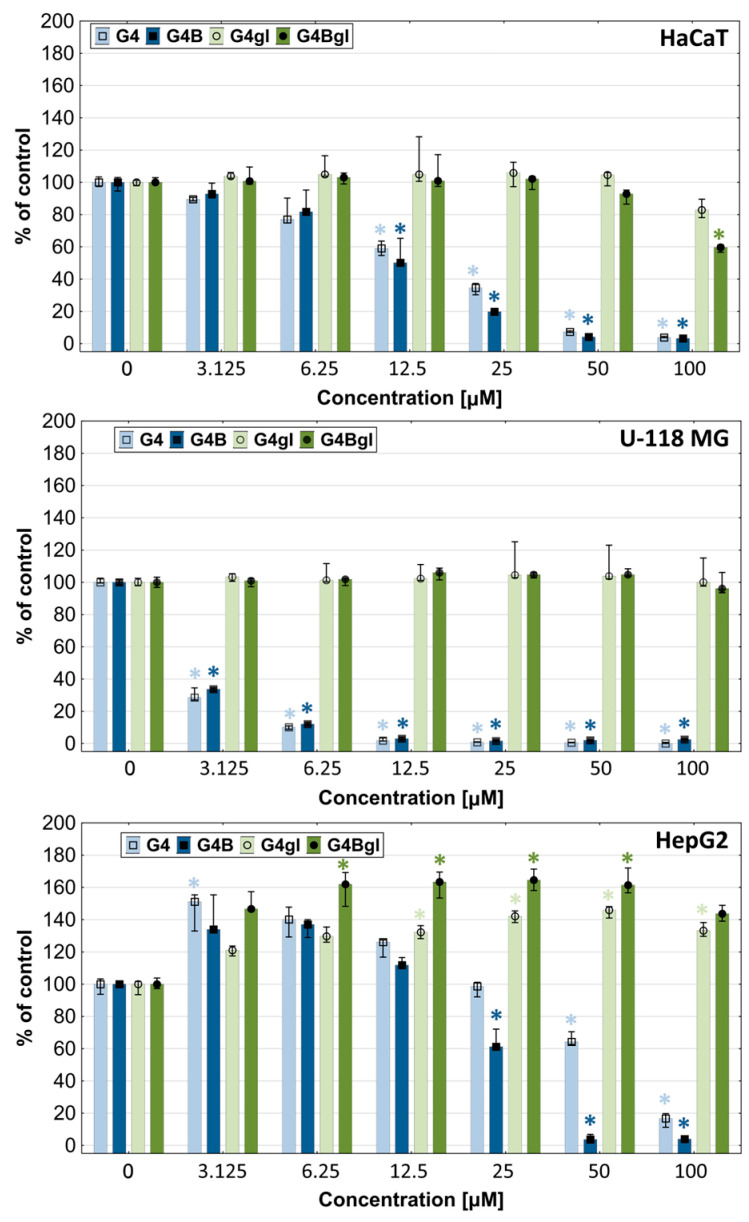
Cytotoxicity of G4, G4B, G4gl, and G4Bgl dendrimers against HaCaT, U-118 MG, and HepG2 cells after 48 h incubation, estimated with an XTT assay. Cell viability is expressed as median of a percent against non-treated control (control expressed as 100%). The whiskers are the lower (25%) and upper (75%) quartile ranges. * *p* ≤ 0.05; Kruskal–Wallis test (against non-treated control).

In order to determine the range of toxic and non-toxic concentrations of the tested PAMAM dendrimer conjugates, a tetrazolium salts assay (XTT) to estimate the mitochondrial oxidoreductase activity was performed. The results show that after 48 h of incubation, the most toxic was the native PAMAM G4 dendrimer, which caused a significant decrease in the viability of HaCaT, U-118 MG, and HepG2 cells from the concentrations of 12.5, 3.125, and 50 µM, respectively. The obtained data are consistent with other research [29]. The biotinylated PAMAM G4B dendrimer was slightly less toxic; however, in HepG2 cells, it exerted a significantly stronger effect than that of native G4 (Figure 4). 

**Figure 5:**
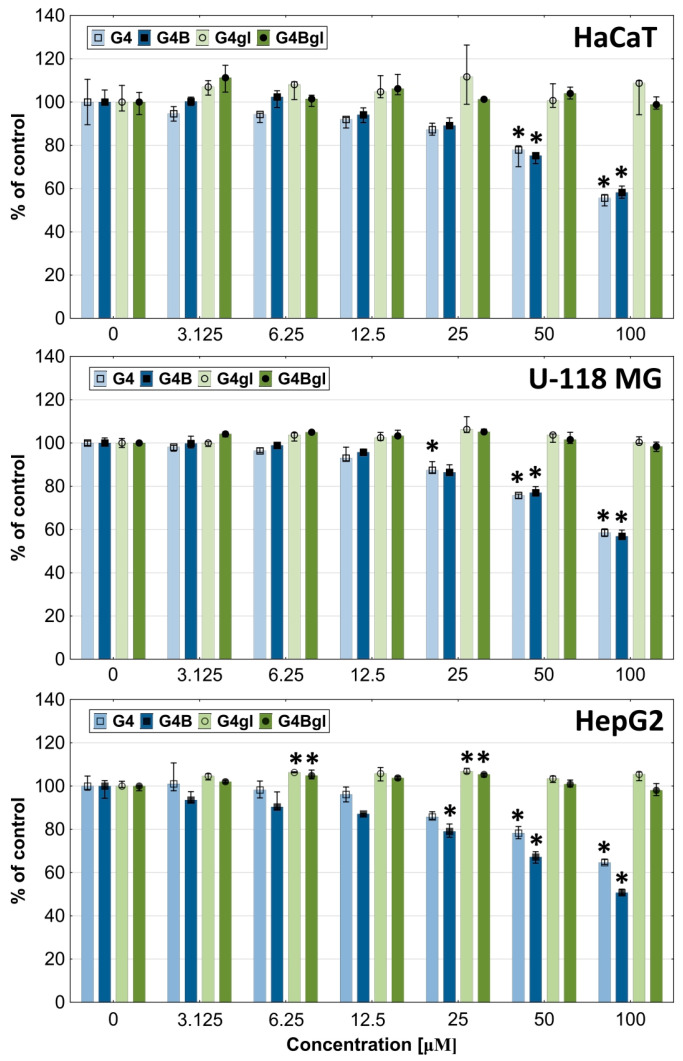
Influence of G4, G4B, G4gl, and G4Bgl dendrimers on HaCaT, U-118 MG, and HepG2 cell proliferation after 48 h incubation. Cell viability is expressed as median of a percent against non-treated control (control expressed as 100%). The whiskers are the lower (25%) and upper (75%) quartile ranges. * *p* ≤ 0.05; Kruskal–Wallis test (against non-treated control).

To assess the viability of cells exposed to the tested compounds, we also used an assay estimating the degree of cell proliferation. Both assays determine the degree of toxicity of the studied dendrimers, but they show differences in their mechanisms. The death of HaCaT and U-118 MG cells occurred mainly through mitochondrial damage induced by G4 and G4B, but not through inhibition of cell division. The antiproliferative effect was visible only at higher concentrations (<25 μM) in all tested cell lines (Figure 5).

Glycidylated and/or biotinylated PAMAM G4 dendrimers were highly biocompatible compared to G4 and G4B. Only HaCaT cells indicated a significant lowering of cell viability after incubation with a 100 µM concentration of G4Bgl (for about 40%). The increase in mitochondrial oxidoreductase activity in hepatocellular carcinoma after incubation with G4gl and G4Bgl (from 3.125 to 100 µM) or G4 and G4B (from 3.125 to 12.5 µM) was interesting. In order to determine whether the increase in viability was related to the growth in the number of cells (proproliferative effect) or the activity of oxidoreductases, a proliferation assay with DNA quantification was performed. It showed that the antiproliferative effect of the tested factors (G4 and G4B) was weak and visible mainly at concentrations of 50 and 100 µM. Meanwhile, the increase in the viability of HepG2 cells was not related to the higher degree of proliferation, but to the effect of the increase in the activity of mitochondrial oxidoreductases (Figure 5).

The presented results indicate that the glycidylation of PAMAM G4 dendrimers (biotinylated or non-biotinylated) significantly increases their biocompatibility. This is consistent with the results obtained for PAMAM dendrimers of the second and third generations [59]. Therefore, the G4gl and G4Bgl dendrimers can be used as carriers of biologically active substances.

### 2.4. In Silico Biotin Binding Site Mapping

**Figure 6:**
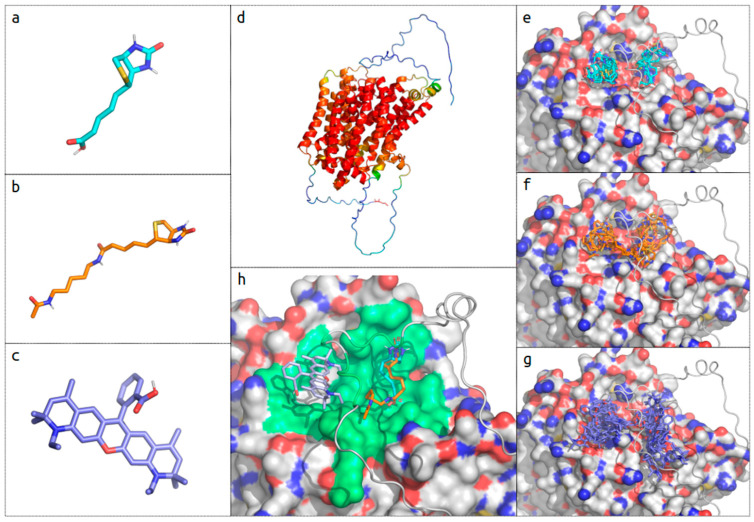
Ligand structures visualization panels (**a**–**c**): native biotin, modified biotin part ATTO 590, and ATTO 590 fluorophore. (**d**) AlphaFold model structure overview (AlphaFold ID: AF-Q9Y289-F1-model_v4); blue regions of the model represent structure regions with low pLDDT score (<50); SMVT N-terminal, bottom and C-terminal, top of the panel, respectively. Docking results for tested ligand panels (**e**–**g**) (ligands color coding as in (**a**–**c**)): the top of the transporter is represented as a surface in which the variable loop region appears as a cartoon in grey (oxygen atoms in red, nitrogen in blue); identified ligand poses are presented in stick representation. (**h**) Comparison of the selected, representative, binding mode for the biotin and the components of the ATTO 590 probe; in green, extended binding surface covering possible binding sites for ligands. Results visualized with open source build of PyMOL (version 3.0.0).

In order to validate the visualization of the biotin uptake experiments, in silico docking simulations were performed. The ATTO 590 biotin probe used contains two moieties: modified biotin and fluorophore (Figure 6a–c).

The simulations were performed for each of them separately and the locations of the putative binding sites were compared with those obtained for the unmodified biotin. The SMVT receptor model structure used in the experiments was obtained from the AphaFold Protein Structure Database. The overall model structure is of good quality, with a pLDDT score well above 70 (Figure 6d). However, both N- and C-terminal regions are of low quality due to the lack of suitable experimental models for this class of molecular transporters. The binding interface analyzed in the simulation was localized at the C-terminal end of the transporter. The interface is defined by the top of the transmembrane ɑ-helix bunch and from the top covered by an extensive loop region (low accuracy score). The obtained results indicate that all of the tested ligands localize in two major putative binding sites (Figure 6e–g). The locations are similar for all analyzed ligands and occupied by a comparable number of obtained ligand poses. In fact, the obtained energy scores for 40 poses for biotin differ between the best and the worst by only about 11%. A detailed analysis shows that a comparable fit can be found in the obtained docking results for the native and modified biotin. Furthermore, in this configuration, the ATTO 590 fluorophore can also be accommodated in functional configuration (Figure 6h). The identified binding sites are part of the interconnected binding surface that can potentially satisfy geometric and physico-chemical requirements from ligands of various sizes and properties.

The binding of native and ATTO 590-modified biotin is comparable in terms of binding pocket localization. Both demonstrate affinity to similar putative binding pockets that are fragments of a larger binding surface able to accommodate the fluorophore of ATTO 590. We conclude that ATTO 590-modified biotin can also be used in biological experiments.

### 2.5. Biotin Uptake

**Figure 7:**
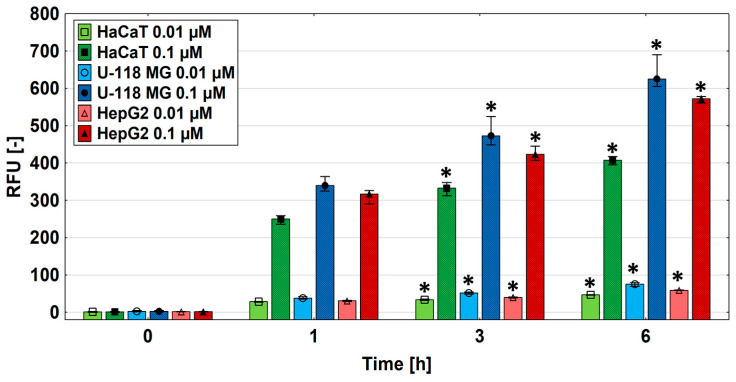
Time-dependent pattern of fluorescently labeled biotin uptake into HaCaT, U-118 MG, and HepG2 cells. Biotin was administered at concentrations of 0.1 or 0.01 µM and incubated for 1, 3, or 6 h. Results are presented as medians; whiskers indicate first and third quartiles. A significant increase in fluorescence was assessed with the Kruskal–Wallis test (* *p* < 0.05).

**Figure 8:**
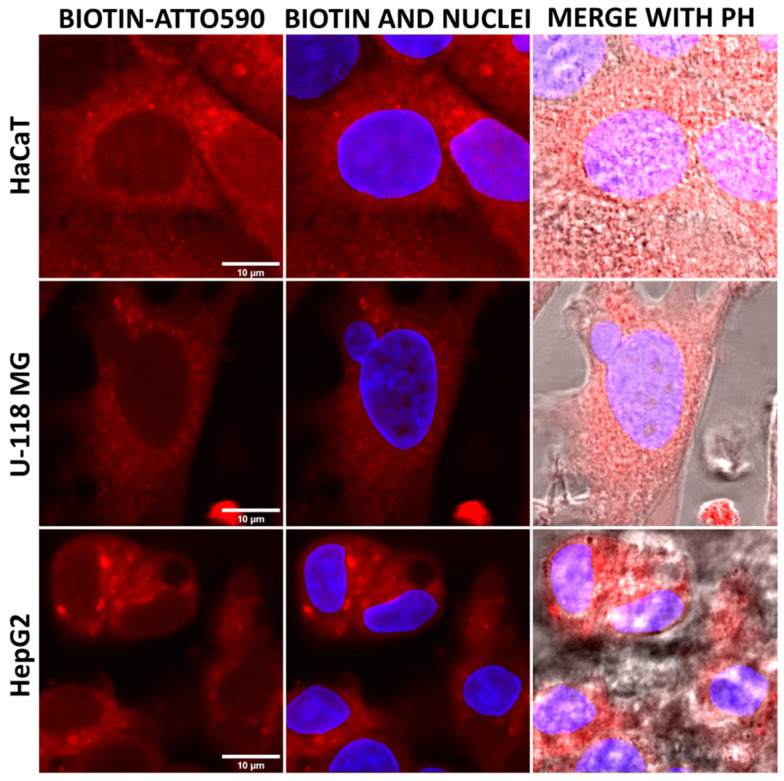
Images from a confocal microscope presenting HaCaT, U-118 MG, and HepG2 cells after 6 h incubation with ATTO 590-labeled biotin at 0.1 µM concentration. Red signal comes from ATTO590, blue from DAPI (nuclei), and PH is phase contrast. Scale bar is equal to 10 µm.

At this stage, we decided to check whether biotin uptake would be directly proportional to the amount of SMVT in the tested cells. For this purpose, fluorescently labeled biotin with ATTO 590 dye was used. Molecules of <1000 Da molecular weight are categorized as small molecules [64]; therefore, ATTO 590-biotin meets the molecular size requirements necessary for transport by SMVT (c.a. 1 kDa). Cells were incubated for 1, 3, or 6 h in 0.01 or 0.1 µM biotin solutions. The results of fluorescence level measurements showed that U-118 MG glioma cells took up fluorescently labeled biotin the most efficiently at both concentrations used. Hepatocellular carcinoma cells showed slightly lower and keratinocytes showed the lowest efficiency. Extending the incubation time resulted in increases in the observed differences between cell types (Figure 7 and Figure 8). 

It can, therefore, be concluded that the cells did not show uptake proportional to the amount of SMVT estimated by the Western blot technique; however, both cancer cell lines with increased levels of SMVT indicated a greater uptake of biotin compared to keratinocytes. Moreover, it seems that the biotin transport was directly proportional to the concentration of biotin. The difference in fluorescence intensity between 0.01 and 0.1 µM biotin concentrations was approximately 10-fold. Images of studied cells collected after 6 h incubation with fluorescently labeled biotin revealed the presence of a significant amount of biotin in HaCaT, U-118 MG, and HepG2 cells in the cytoplasmic area (in vesicles and cytoplasm) and also in the space of the cell nuclei. The biotin accumulation sites, visible as red dots of higher signal intensity, may represent mitochondria since it is known that biotin is actively transported there by the monocarboxylate transporter (MCT-1) into these organelles [65]. 

### 2.6. Time-Dependent Dendrimer Uptake

Nowadays, modified PAMAM dendrimers are considered drug carriers due to their good biocompatibility and biodegradability. They can improve the solubility and reactivity of drugs entrapped in their internal cavities or attached to surface functional groups [66]. PAMAM dendrimers can enhance drug storage stability and drug biodistribution as well as increase permeation and retention for targeting tumors. Their properties were confirmed by numerous studies using them, for instance as carriers for paclitaxel [67] or methotrexate [68]. We proposed using hydroxylated and/or biotinylated PAMAM G4 dendrimers as drug vehicles since Zhang et al. showed that hydroxylation of PAMAM dendrimers decreased the exocytosis (efflux) of PAMAM dendrimers compared to cationic and anionic ones. Hydroxylated PAMAMs were also not affected by the major vault protein (MVP) responsible for drug resistance in some cancer cells [69]. Additionally, we wanted to further reduce the resistance and efflux of dendrimers by biotinylation, since it is known that biotin-mediated endocytosis may overcome tumor drug resistance through biotin receptor-mediated endocytosis [70].

**Figure 9:**
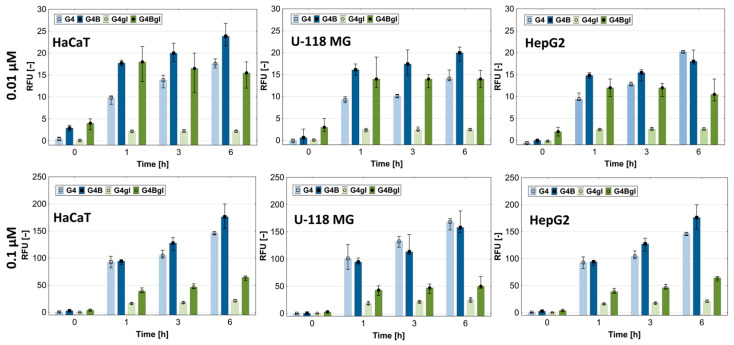
Time-dependent uptake efficiency of the tested PAMAM dendrimers G4, G4B, G4gl, and G4Bgl by HaCaT, U-118 MG, and HepG2 cells. Cells were incubated for 1, 3, or 6 h at 0.01 or 0.1 µM concentrations of the tested compounds. Results are presented as medians; whiskers indicate first and third quartiles.

Biotinylation of PAMAM G4 dendrimers (positively charged or neutral) was supposed to increase their uptake, especially by SMVT overexpressing cancer cells. Incubation of the tested cells at a 0.1 µM dendrimer concentration resulted in the active uptake of all tested conjugates. The most efficient uptake was observed in the case of cationic dendrimers, with the amount of biotinylated and native dendrimers being similar in all cell types. A slightly higher uptake of biotinylated G4B dendrimers was visible from the third hour of incubation in HaCaT and HepG2 cells, while glioma took up native dendrimers more efficientlyIn the case of hydroxyl-terminated G4gl dendrimers, biotinylated derivative (G4Bgl) was taken up 2–3 times more efficiently than their non-biotinylated analog. However, biotinylated and glycidylated G4Bgl derivatives were always about 2–3 times less absorbed than their biotinylated forms terminated with amino residues (G4B) (Figure 9).

The situation changed significantly after using the tested dendrimers at a lower, 0.01 µM, concentration. The G4B was taken up significantly more efficiently than G4 in all cell lines (by about 20–40%), while the G4Bgl cellular intake was about five times stronger than that of G4gl. At the same time, G4Bgl reached the level of native dendrimer uptake (Figure 9). It was described that the biotin transporter SMVT tended to have a Michaelis–Menten constant (km) in the low- to mid-micromolar range, but some biotin conjugates were effective in targeted delivery in the nanomolar range [71].

Interactions between PAMAM dendrimers and cell membranes remain poorly understood; however, it is suspected that PAMAM cytotoxicity is caused by the interaction of their cationic residues with negatively charged cell membranes. As a result, nanopores are formed in the latter, through which dendrimers penetrate into the cells, while damage to the membrane causes leakage of cellular content, and eventually cell death [29,72]. A 10-fold decrease in the concentration of the PAMAM G4 dendrimer probably resulted in a reduced effect on the membranes of the tested cells and, in consequence, in a weaker penetration of these nanoparticles into the cells. Meanwhile, the uptake of biotinylated analogs was still productive, which means that G4B was taken up more efficiently than native PAMAMs.

OH-terminated G4gl dendrimer penetration of the tested cells was significantly weaker. This is consistent with the observations of Liaw et al. They show that PAMAM G4 OH-terminated dendrimer uptake was lower in BV2 microglia cells than native G4 amino-terminated PAMAM dendrimers [73]. Also, Perumal et al. proved, in the A549 cell line model, that cationic G4 PAMAM amino-terminated dendrimers were taken up more efficiently than OH-terminated analogs [74]. The biotinylation of the glycidylated dendrimer increased its uptake much more, due to the fact that G4gl penetrated into cells at a very low rate. This leads to the conclusion that biotinylated dendrimers should be used in targeted therapy at very low concentrations (<0.01 µM), so that they are taken up most efficiently and do not unintentionally penetrate into other, healthy cells.

**Figure 10:**
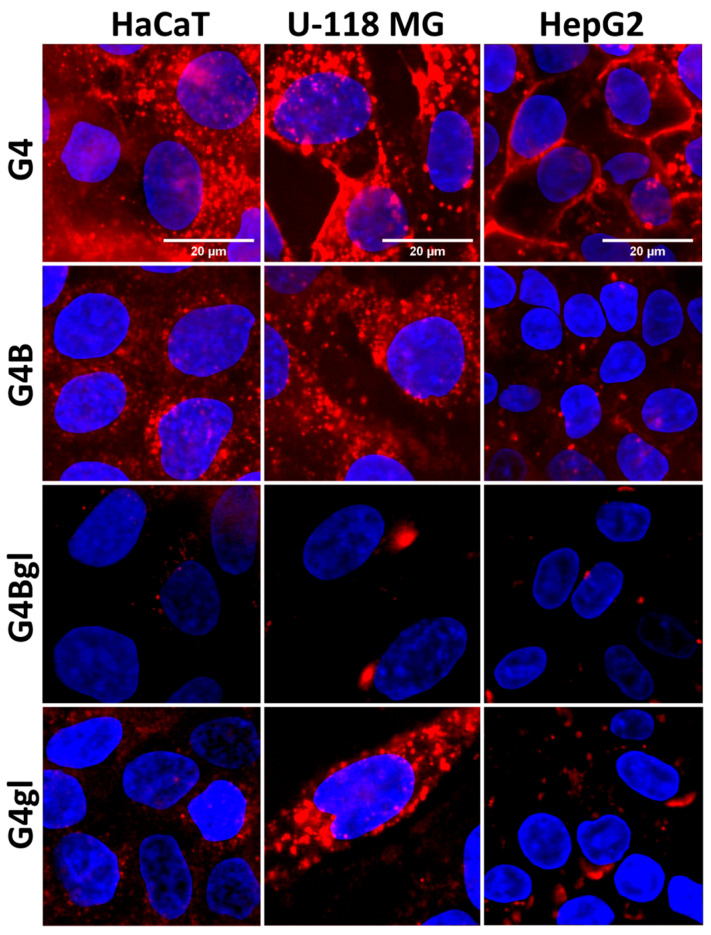
Images from confocal microscope showing the uptake efficiency of 0.1 µM solutions of PAMAM dendrimers G4, G4B, G4gl, and G4Bgl by HaCaT, U-118 MG, and HepG2 cells after 6 h incubation. Red signal comes from dendrimers labeled with Cy5, blue from DAPI-stained nuclei.

For all the cells tested, biotinylation increased the cellular uptake of dendrimers. In HepG2 cells, the level of dendrimers was the lowest, while in human keratinocytes, it was the highest. The lower level of dendrimers in HepG2 cells can be explained by the efflux phenomenon. As can be seen in Figure 10, PAMAM G4 was localized mainly in the cellular membrane of HepG2 cells, but not inside the cells. A weaker effect was seen in the case of G4B (Figure 10). 

A similar effect was described by Zhang et al., who observed in drug-resistant breast cancer cells (MCF-7/ADR) that the exocytosis rate was the highest for PAMAM-NH_2_ and the lowest for PAMAM-OH [69]. This phenomenon can be related to the presence of cell-surface major vault protein (MVP) in the cellular membrane of HepG2 cells [75]. HaCaT cells internalized studied dendrimers (0.01 µM) the most efficiently, especially biotinylated G4B and G4Bgl (Figure 9 and Figure 10). It is known that this cell line not only shows a high degree of SMVT expression but also has another, additional biotin transport mechanism, probably related to MCT-1 [61,71]. However, uptake studies using biotin analogs having modified carboxylic acid such as biotin methyl ester and a-lipoic amide did not result in a marked decrease in biotin uptake by intestinal cells [76]. Mitra et al. showed that SMVT in retinoblastoma cells does not interact with biotin analogs having a modified carboxyl group [77]. Finally, Tripathi et al. in a review reassumed that the presence of a free aliphatic carboxylic acid group is the only common feature that exists among these structurally diverse substrates of SMVT. However, many authors have used various drug–biotin conjugates synthesized through amidation or esterification of the biotin carboxylic acid group and have demonstrated an increased uptake of such compounds [71]. 

As shown above in Figure 7, the uptake of fluorescently labeled biotin was higher in cancer cells than in keratinocytes. Unfortunately, the transport of biotinylated G4B and G4Bgl dendrimers was not directly analogous to the biotin penetration profile or to the SMVT level in the studied cells. There may be several explanations for this phenomenon. Many researchers assume that biotinylated nanoparticles (including dendrimers) may potentially enter cells via SMVT-related transport. This seems unlikely, given the fact that many biotinylated nanoparticles are too large to penetrate through SMVT (>1 kDa), since the tested dendrimers were about 14–20 kDa. However, they could enter cells by endocytosis, and all types of endocytosis could be used for this purpose. Chen et al. proved, using specific inhibitors of clathrin-mediated endocytosis, micropinocytosis, and caveolae-mediated endocytosis, over 50%, 15%, and 30% decreases in biotinylated chitosan surface-modified poly (_D,L_-lactide-*co*-glycolide) nanoparticle (Bio-PLGA NPs) uptake [78]. Micropinocytosis inhibitor (amiloride) may also have an inhibitory effect on biotin transport via SMVT, since it can block the Na^+^ channel [79]. Similarly, biotinylation of the studied PAMAM dendrimers increased their uptake, indicating a biotin receptor-dependent endocytosis process: they were 2–5 times more efficiently transported into cells (Figure 9), microscopic images showed the existence of endocytic vesicles (Figure 10) in contrast to the images for biotin itself, and their size was too large to be transported by SMVT or even MCT. So far, a biotin receptor responsible for inducing endocytosis has not been identified, but its existence is postulated on the basis of results from many publications [71]. The obtained results show the different mechanisms that were involved in the interaction of biotin and biotin conjugates with their specific uptake systems; this needs more attention.

### 2.7. Dendrimer Accumulation

**Figure 11:**
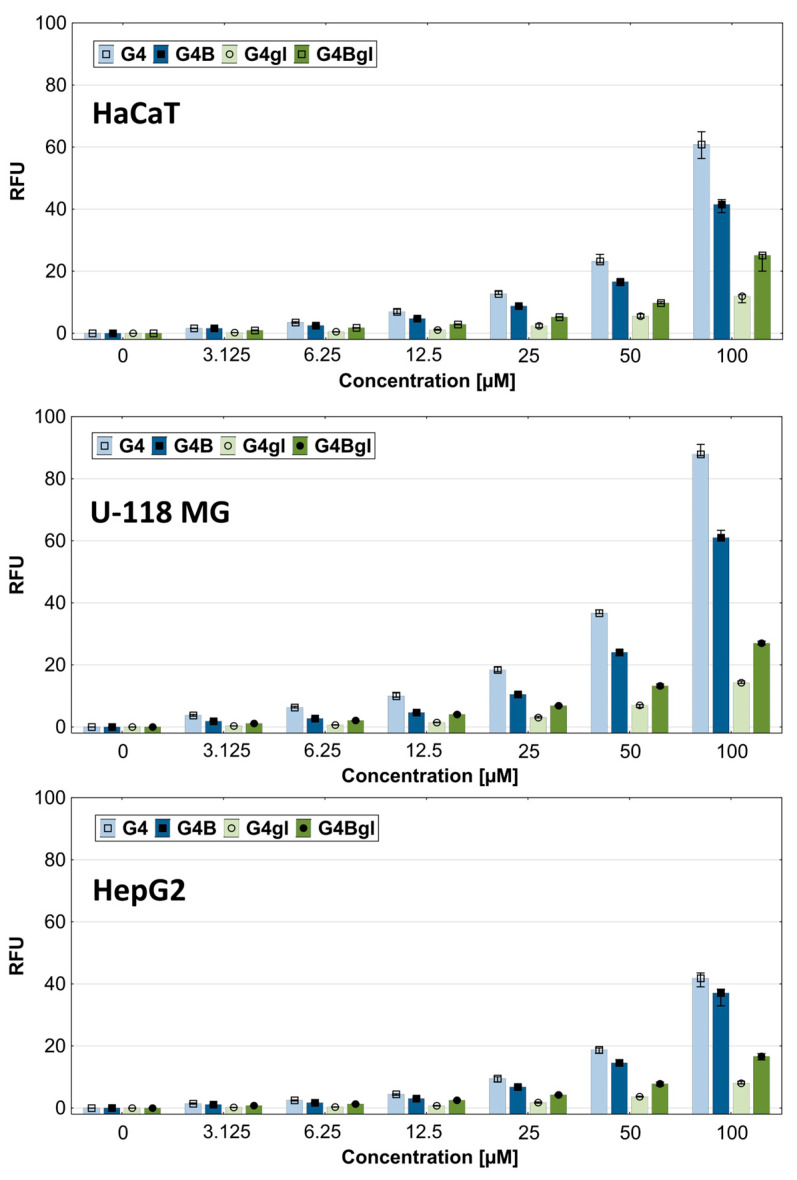
Accumulation of G4, G4B, G4gl, and G4Bgl dendrimers after 48 h incubation in the range of 3.125–100 µM concentrations. Results are presented as medians; whiskers indicate first and third quartiles.

In addition to time-dependent uptake, it is very important to examine the cellular accumulation of potential drug carriers after long-term incubation, in a wide range of concentrations. Therefore, we examined the degree of dendrimer accumulation after 48 h of incubation in the concentration range from 3.125 to 100 µM. With increasing concentration, the degree of cellular content of all dendrimers increased. The results show that the G4 dendrimer accumulated in cells to the highest extent, while G4B accumulated to a significantly lower one. This was unlike the result in HepG2 cells, where the amount of G4B was similar to that in HaCaT cells, but the accumulation of native G4 was not significantly higher. This may confirm the existence of the G4 efflux phenomenon observed and described above (Figure 10). This may also be evidenced by the observed increase in mitochondrial oxidoreductase activity, which suggests that these cells required higher energy inputs for efflux (Figure 4). It was probably similar in the case of the other dendrimers, which resulted in the fact that HepG2 cells accumulated the tested compounds to the lowest extent, especially compound G4 (Figure 11).

**Figure 12:**
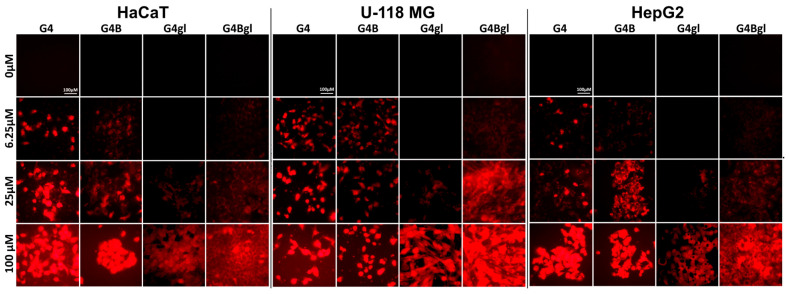
Images from fluorescence microscope showing the accumulation of G4, G4B, G4gl, and G4Bgl PAMAM dendrimers in HaCaT, U-118 MG, and HepG2 cells after 48 h incubation at 6.25–100 µM concentrations. Scale bar is equal to 100 µm.

The least amount of the G4gl dendrimer hydroxylated with glycidol was observed, while its biotinylated analog level was significantly increased in the tested cells (approximately two times). It is also worth noting that glioma cells absorbed the largest amount of the tested dendrimers compared to the other cell lines (at the highest concentration of 100 µM even by 50%). This was related to the lowering viability of these cells under the influence of dendrimers, the decrease in which was visible already from the lowest concentration of 3.125 µM (Figure 4). This was confirmed by fluorescence microscope images, which showed the highest fluorescence intensity in glioma cells. It is also worth looking at the distribution of dendrimers in cells. It can be seen that the amount of G4 and G4B dendrimers in cells was closely correlated with their viability and was the lowest in the most resistant HepG2 cells, where only single cells contained significant amounts of dendrimer (Figure 12).

Biotinylated dendrimers were best visible in glioma cells, and slightly weaker in HaCaTs. Both G4 and G4B were able to penetrate cell nuclei. In the case of G4gl, however, it penetrated efficiently into the nuclei of HaCaTs and U-118 MG cells, but not all HepG2 cells, which again confirms the occurrence of the reverse efflux phenomenon. Hepatocellular carcinoma is considered to be highly resistant to many drugs [16]. The G4Bgl dendrimer penetrated efficiently into the nuclei of U-118 MG glioma cells only (Figure 12), despite the lack of impact on the viability of these cells (Figure 4). This property means that this conjugate can be a selective carrier of genes and drugs even to the cells whose receptors are located in the glioma cell nuclei. 

### 2.8. Toxicity against C. elegans

A very important issue is to determine the toxicity of potential drug carriers and therapeutic agents not only in vitro but also in vivo. For this purpose, we chose the model of the nematode *C. elegans*—a non-pathogenic invertebrate. The *C. elegans* toxicity assays provide data concerning a whole animal with intact and metabolically active digestive, reproductive, endocrine, sensory, and neuromuscular systems. *C. elegans* is an intermediate model between in vitro and mammalian testing, and toxin ranking using various *C. elegans* assays has consistently predicted toxicity ranking in mammals [80]. Moreover, results of studies indicate significant parallels, not only regarding dose additivity, but also in terms of potency assessment of substances, between *C. elegans* and the regulatory standard study conducted in rats, which is currently the gold standard for toxicity testing [81].

**Figure 13:**
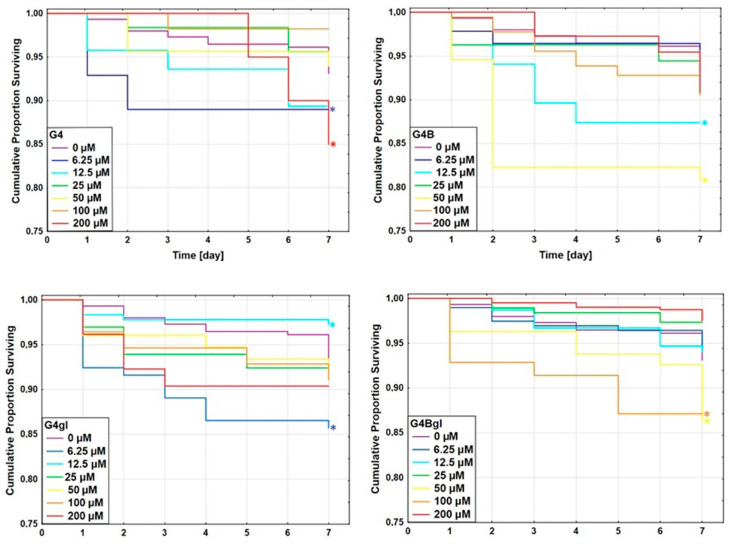
The Kaplan–Meier survival curves of *C. elegans* after 7 days of incubation with G4, G4B, G4gl, and G4Bgl. Results are presented as cumulative proportion surviving. Statistically significant differences against control obtained in Gehan’s Wilcoxon test are marked with asterisks * (*p* < 0.05) in the colors corresponding to the tested concentrations.

The viability of *C. elegans* was evaluated following a seven-day incubation period with G4, G4B, G4gl, and G4Bgl dendrimers. Studies confirmed the low toxicity of the considered conjugates. Both G4 alone and glycidylated and/or biotinylated PAMAM dendrimers caused a low mortality of nematodes. The highest mortality was already observed for 6.25 μM G4gl after the first day of incubation. This did not change by the end of the study despite the fact that for the last 3 days of the test, there was no change in fatality. For this dendrimer, the supreme decrease in viability was close to 15%. The biotinylated dendrimer (G4B) also revealed one concentration that affected the nematode viability the most—25 μM. The highest increase in mortality was observed after the second day of incubation with the carrier. The concentration that also changed the viability more than others was 12.5 μM—here, the final decrease in viability reached about 12.5%. Overall, the greatest decrease in viability in all cases was observed for the 50 μM biotinylated dendrimer, which was less than 20%. The highest analyzed concentration was the most toxic for native PAMAM G4 without any modifications. The concentration of 6.25 μM resulted in similar cytotoxicity for G4 and G4gl. For G4Bgl, the highest mortality was induced by the concentration of 100 μM (Figure 13).

**Figure 14:**
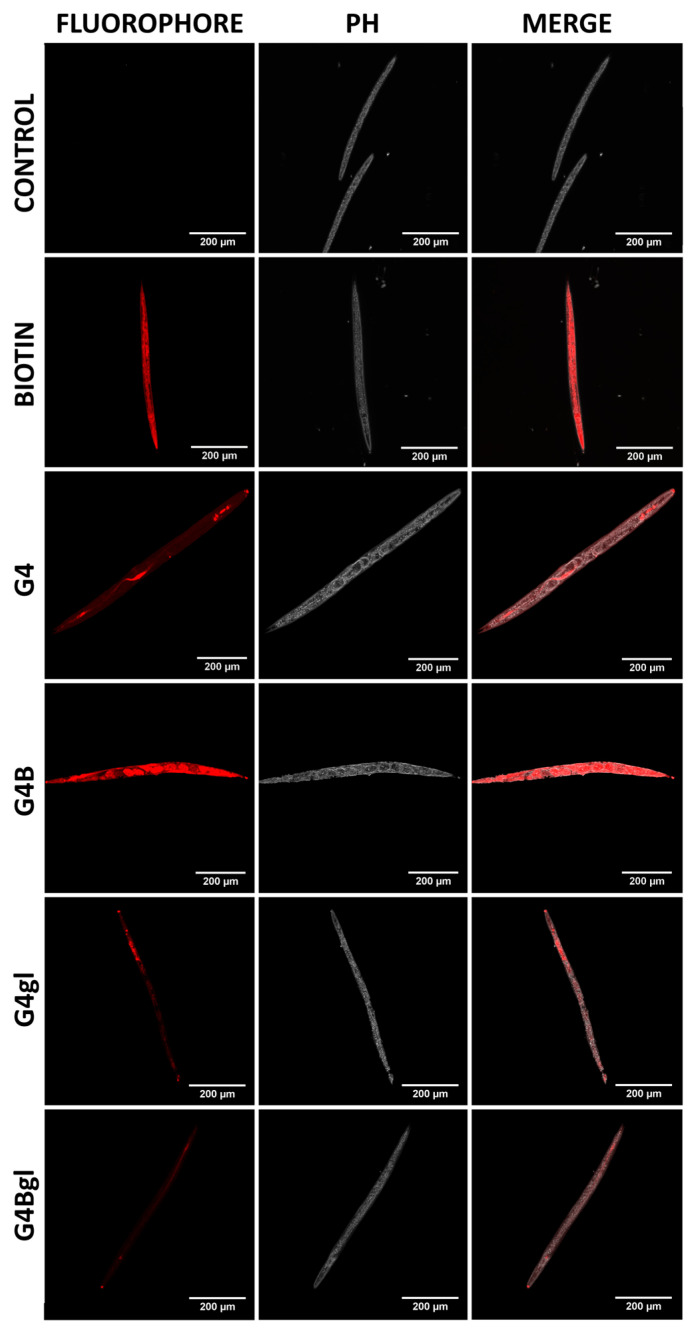
Images from confocal microscope, presenting the fluorescently labeled biotin or G4, G4B, G4gl, and G4Bgl dendrimer contents in *C. elegans* body after 7-day incubation with 6.25 µM solutions (red signal). PH–phase contrast. Scale bar = 200 µm.

A little more light on the usefulness of the tested dendrimers in vivo is shed by microscopic observations showing the content of conjugates and fluorescently labeled biotin inside the body of *C. elegans* after a 7-day incubation at concentrations of 6.25 µM. They showed that the nematodes actively accumulated biotin, and its content was observed throughout the body. Meanwhile, the native G4 dendrimer was internalized into the body cells in trace amounts, and its greater amount was detected in the lumen of the nematode digestive tract, similar to G4gl and G4Bgl. Only the biotinylated G4B dendrimer penetrated very efficiently into all cells of the nematode body, and its highest amount was observed in hermaphrodite embryos (Figure 14). 

There are some studies [9,10] that show biotin/biotin ligation protein in *C. elegans* as crucial elements that can affect embryo/larval development. This can indicate biotin and, related to it, proteins as particles/components that have an important influence on the nematode life cycle. Based on our results, it can be said that biotin-furnished dendrimer at the concentrations we tested is harmless and does not significantly reduce the lifetime of *C. elegans*. A conclusion can be that biotin in adulthood, when the nematode is developed, is not a factor that significantly affects mortality. The obtained results may also indicate that biotinylation of G4 significantly increases the efficiency of its transport into the *C. elegans* body. The lack of such an effect on G4gl can be explained by the fact that hydroxylated nanoparticles use other types of transmembrane transport (endocytosis) or by the reverse efflux of these nanoparticles. Therefore, in addition to the fact that the tested dendrimers were highly biocompatible in vivo, G4B may be a carrier of potential anti-nematode drugs, increasing their uptake, accumulation, and efficacy.

### 2.9. Penetration into the Rat’s Brain

**Figure 15:**
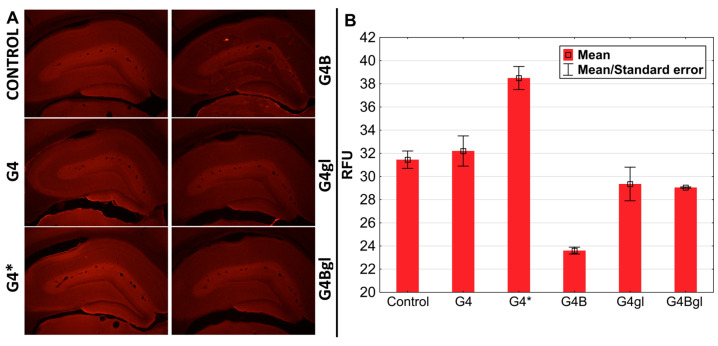
(**A**) Images from fluorescence microscope, presenting fluorescence signal in area of the Wistar rat’s hippocampus after Cy5-labeled G4, G4B, G4gl, and G4Bgl dendrimers’ administration at 16 nmol/g.b.w. (**B**) Mean grey values in the hippocampus area estimated with ImageJ 1.49v software.

Dendrimer accumulation studies showed that their level was the highest in glioma cells (Figure 11). Therefore, in the next step, the ability of the tested PAMAM conjugates to penetrate the central nervous system (including the brain–blood barrier) of Wistar rats was examined. Analyses of fluorescence intensity in the hippocampus area showed that the Cy5-labeled dendrimers in the low dose of 16 nmol/g.b.w. used did not cause an increase in fluorescence intensity. Only the G4* dendrimer administered in a double dose (32 nmol/g.b.w.) was detected in the hippocampus area (Figure 15).

After the administration of the other conjugates used in the basic concentration, a decrease in fluorescence intensity was observed compared to the untreated control, which also indirectly confirms their presence in the hippocampus area. PAMAM nanoparticles, especially those modified with glycidol and/or biotin, might exhibit the phenomenon of fluorescence and, related to it, quenching [82]. The addition of biotin (also a fluorescently active molecule) and glycidol can additionally shift the absorption band of the obtained conjugates. It has been shown that the presence of biotin in the solution can significantly reduce the emission spectra’s intensity, for instance, a sample of ZnS:Mn [83]. In our experiment, the fluorescence of the rat’s hippocampus was the lowest after the administration of the conjugate with biotin (G4B) (Figure 15). This phenomenon needs more in-depth studies.

In our experiment, we used probably too-low doses of dendrimers, and additionally, their labeling with the fluorescent marker Cy5 was too low (about 20% of the dendrimer molecules were substituted with the fluorophore). Due to the lack of a larger amount of conjugates, we could not repeat the experiment. We were, therefore, unable to demonstrate the ability of the tested conjugates to penetrate the brains of Wistar rats. The observation of the presence of the PAMAM G4* dendrimer revealed at a dose of 32 nmol/g.b.w. in the rat’s CNS is consistent with the literature data, because it has been shown that G4 PAMAM dendrimers and biotinylated G4 PAMAMs can penetrate into the rat’s brain (striatum) [49]. Also, hydroxylated PAMAM G4 and G6 dendrimers were shown to be capable of penetrating the brains of 9L rats [84]. This shows that our conjugates should also exhibit such abilities and can be considered as suitable carriers to efficiently transport drugs into glioma cells in the CNS area. It is worth emphasizing that the administration of three doses of the tested conjugates to rats did not cause any negative effects on their behavior.

## 3. Materials and Methods

### 3.1. Materials

Biotin N-hydroxysuccinimide ester (biotin-NHS) and common chemicals were purchased from Merck (KGaA, Darmstadt, Germany). Sulfo-cyanin5 succinimidyl ester was purchased from MedChemExpress (Sollentuna, Sweden). Spectra/Por^®^ 3RC dialysis membrane (cellulose, MWcutoff = 3.5kDa) was provided by Carl Roth GmbH&Co KG (Karlsruhe, Germany). Eagle’s Minimum Essential Medium (EMEM) was purchased from ATCC (Manassas, VA, USA). Dulbecco’s Modified Eagle’s Medium (DMEM) and fetal bovine serum (FBS) were provided by Corning Incorporated (Corning, NY, USA). Trypsin–EDTA solution was purchased from Capricorn scientific (Ebsdorfergrund, Germany). Dulbecco’s phosphate-buffered saline (PBS) without magnesium and calcium ions and DAPI (4′,6-diamidino-2-phenylindole, dihydrochloride) were from Thermo Fisher Scientific (Waltham, MA, USA). PBS was provided by Roth (Karlsruhe, Germany). XTT sodium salt (2,3-bis [2-methoxy-4-nitro-5-sulfophenyl]-2Htetrazolium-5- carboxanilide inner salt) was from Cayman (Ann Arbor, MI, USA). Phenazinemethosulfate (PMS), N-methyl dibenzopyrazine methyl sulfate, penicillin and streptomycin solution, 0.4% trypan blue solution, and Atto 590-Biotin BioReagent were purchased from Sigma-Aldrich (St Louis, MO, USA). All reagents for electrophoresis and Western blotting were provided by Bio-Rad (Hercules, CA, USA). Cell culture dishes were from Corning Incorporated (Corning, NY, USA) and Nunc (Rochester, NY, USA).

### 3.2. Methods

The 1H NMR spectra were recorded in deuterated water using Bruker 300 MHz (Rheinstetten, Germany) and worked up with TopSpin 3.5 software at the College of Natural Sciences, University of Rzeszów.

### 3.3. Syntheses

Polyamidoamine dendrimer of fourth generation (G4) was synthesized on the 0.5 mmole scale according to the original procedure described by Tomalia et al. [55] and characterized by the 1H and 13C NMR spectroscopy as was published recently [56]. It was further used for fluorescent labeling and functionalization with biotin and glycidol.

In particular, 649 mg of G4 (42.2 µmoles) in 19 mL DMSO was reacted with 32 mg Cy5SE-NHS (sulfo-cyanin5 succinimidyl ester, 42 µmoles) for 24 hr at room temperature. Afterwards, the reaction mixture was transferred into a cellulose dialytic tube and dialyzed against water for 48 h in the dark until the receiving water was colorless. Then, a product was isolated from the tube, water was removed in vacuo by rotary evaporation, and the level of substitution was determined by ^1^H NMR spectroscopy (20% labeling). The UV-Vis spectrum of the product was taken in order to confirm the level of labeling based on the absorption value at 646 nm corresponding to a strong band (extinction coefficient = 2.7 × 10^5^). A viscous resin of G4 was isolated (614 mg, 40 µmoles, 94% yield). 615 mg of viscous resin of G4* was isolated (40 µmoles, 94% yield). The obtained G4* was further used to conjugate with biotin and to eradicate terminal amine groups of G4 by the addition of R-glycidol as follows.

142 mg of G4* (10 µmoles) was dissolved in methanol (5 mL). R-glycidol (190 mg, 2.56 mmoles) was added and the mixture was reacted for 48 h at room temperature. The mixture was dialyzed against water for 3 days in order to remove unreacted glycidol. The isolated product was identified by ^1^H NMR spectroscopy as G4g* with primary amine groups of G4 totally double-substituted with R-glycidol (128 2,3-dihydroxypropyl groups). Yield: 201 mg (8.4 µmoles, 85%).

284 mg of G4* (20 µmoles) was dissolved in 4 mL DMSO and 13.7 mg of biotin succinimidyl ester (biotin-NHS, 40 µmoles) was added, and the solution was left at room temperature for 24 h. The product was purified by dialysis in order to remove the released succinimide side product (3 days against water) and isolated, and the stoichiometry was determined by ^1^H NMR spectroscopy as G4B*. Yield: 162 mg (18 µmoles, 90%).

Then, 145 mg of G4B* (10 µmoles) was dissolved in methanol (8 mL) and 190 mg of R-glycidol (2.56 mmoles) was added dropwise. The mixture was reacted for 40 h at room temperature. The product was isolated as before and characterized by ^1^H NMR spectroscopy. The primary amine groups of G4 were totally double-substituted with R-glycidol in G4gB*. Yield: 200 mg (8.4 µmoles, 84%).

All dendrimers, G4, G4g, G4B, and G4gB, are well soluble in water. The stock 7.0 mM solutions were used for biological tests.

### 3.4. Cell Cultures

Human immortalized keratinocytes (HaCaTs) obtained from Cell Lines Service (Eppelheim, Germany) and human glioblastoma cell line (U-118 MG) obtained from ATCC (Manassas, VA, USA) were cultured in DMEM (doubling time 24 and 35 h, respectively). Human hepatocellular carcinoma (HepG2) from the European Collection of Authenticated Cell Cultures (ECACC) (doubling time 48 h) was grown in EMEM. Media were supplemented with 10% heat-inactivated FBS and 100 U/mL penicillin, and 100 μg/mL streptomycin. Cells were incubated at 37 °C in humidified 95% air with 5% CO_2_. Media were changed every 2–3 days and cells were passaged at 70–80% confluence after treatment with 0.25% trypsin–EDTA/PBS (calcium- and magnesium-free). Cell morphology was checked using a Nikon TE2000S Inverted Microscope (Tokyo, Japan) with phase contrast. The number and viability of cells were estimated by the trypan blue exclusion test using an Automatic Cell Counter TC20TM (Bio-Rad Laboratories, Hercules, CA, USA). 

### 3.5. SMVT Expression

HaCaT, U-118 MG, and HepG2 cells were cultured to 70–80% confluence as described above. Harvested cells were lysed with 50 µL per 1 × 10^6^ cells of RIPA Lysis Buffer (Millipore, #20-188) containing protease inhibitors (30 min, 4 °C) and centrifuged (13,000 rpm, 4 °C, 15 min). Western blot analysis was performed by loading 15 μL of cell lysates (containing 3·10^5^ cells) and 5 μL of Precision Plus Protein Dual Color Standards (Bio-Rad, #161-0374) onto a 5% stacking gel. SDS-PAGE was carried out using a 10% resolving gel. After separation, proteins were transferred to Immun-Blot PVDV Membranes for Protein Blotting (Bio-Rad, #162-0177) and blocked in 1% BSA in Tris Buffered Saline with 0.05% Tween 20 (TBST), pH 7.5 for two hours at room temperature on a rocking platform. Sodium dependent multivitamin transporter (SMVT) was detected at 68 kDa using an anti-SLC5A6 polyclonal antibody (Sigma-Aldrich, #SAB4503495) at a dilution of 1:500 in 1% BSA in TBST for two hours at room temperature on a rocking platform, followed by a goat anti-rabbit IgG-HRP secondary antibody (Jackson ImmunoResearch, #111-035-003) at a dilution of 1:5000 for one hour. Colorimetric detection was performed using DAB-buffer tablets (Millipore-Sigma, #EMD1.02924.0001) for 5 min.

### 3.6. Cytotoxicity

Cells were seeded in flat-bottom 96-well plates at a density of 1 × 10^4^ cells/well and allowed to attach for 24 h. Working solutions of dendrimers (3.125–100 µM) in culture media were added into cells. After 48 h exposure to dendrimers, the medium was removed, an XTT mixture of 1.7 mM of XTT and 8.3 μM of PMS in the complete medium was added (100 µL/well), and the plates were incubated at 37 °C for 48 h. Then, the absorbance was measured at 450 and 620 nm against a blank sample (complete growth medium with XTT and PMS), with a microplate reader (μQuant–BioTek, Winooski, VT, USA). The results were expressed as a percentage of the non-treated control.

### 3.7. In Silico Biotin-Binding Site Mapping

Mapping of the putative binding sites was performed using tools provided by the SwissDock server (https://www.swissdock.ch/ accessed on 5 August 2024) using the AutoDock Vina algorithm implementation. In all cases, the search box center was defined at 12, 4, −12 Å and the size of the box kept constant at 30, 30, 30 Å. The SMVT (UniProt ID: Q9Y289) receptor structure was downloaded from the AlphaFold Protein Structure Database without modification (AlphaFold ID: AF-Q9Y289-F1-model_v4). The ligands were represented by canonical SMILES codes obtained from the PubChem database (https://pubchem.ncbi.nlm.nih.gov/ accessed on 5 August 2024) for biotin and ATTO 590 biotin. In the case of ATTO 590 biotin, the SMILES string was divided into two parts containing the modified biotin and fluorophore moiety before performing docking experiments. Results were visualized using an open source build of PyMOL (version 3.0.0).

### 3.8. Time-Dependent Biotin Uptake

Cells were seeded in flat-bottom 96-well plates at a density of 4 × 10^4^ cells/well (100 µL) and allowed to adhere for 12 h. Solutions of ATTO 590 fluorescently labeled biotin (0.01 and 0.1 µM) were prepared in DMEM culture media and added into HaCaT, U-118 MG, and HepG2 cells for 1, 3, or 6 h (100 µL per well). After exposure, the medium was removed, cells were washed with warm PBS, and the fluorescence intensity was measured with an Infinite M200 PRO (TECAN Group Ltd., Männedorf, Switzerland) 580/635 nm (ATTO 590) against a blank sample (medium without cells).

### 3.9. Time-Dependent Dendrimer Uptake

Cells were cultured as described above. Solutions of G4, G4B, G4gl, and G4Bgl fluorescently labeled Cy5 dendrimers at 0.01 and 0.1 µM concentrations in DMEM culture media were added into HaCaT, U-118 MG, and HepG2 cells for 1, 3, or 6 h (100 µL per well). After incubation, the medium was removed, cells were washed with warm PBS, and the fluorescence intensity was measured with an Infinite M200 PRO (TECAN Group Ltd., Männedorf, Switzerland) 640/680 nm (Cy5) against a blank sample (complete medium without cells).

### 3.10. Proliferation and Dendrimer Accumulation

The effect of the tested compounds on cell proliferation was assessed by DNA quantification with DAPI staining. For this purpose, 4 × 10^3^ cells per well were seeded on 96-well plates with a flat bottom and incubated for 24 h at 37 °C to adhere to the bottom of the plate. After the culture medium removal, the cells were treated with working solutions of the tested dendrimer conjugates for 48 h, at increasing concentrations (3.125–100 µM). Then, the culture medium was removed, the cells were fixed in a 3.7% formalin solution, and the nuclei were stained with 600 nM DAPI solution in PBS for at least 1 h. Fluorescence was then measured with an Infinite M200 PRO microplate reader (TECAN Group Ltd., Männedorf, Switzerland) at 360/460 nm (DAPI) against a blank (wells without cells). The number of cells was directly proportional to the fluorescence intensity. Dendrimer accumulation after 48 h incubation was assessed via measurement of Cy5 fluorescence intensity with an Infinite M200 PRO microplate reader (TECAN Group Ltd., Männedorf, Switzerland) at 640/680 nm against a blank (wells without cells). Fluorescence intensity was converted to an equal number of cells by referencing the fluorescence intensity of Cy5 to the signal derived from DAPI.

### 3.11. Toxicity against Caenorhabditis elegans

*C. elegans* was used to estimate the toxicity and accumulation of G4 PMAM dendrimers alone and of the glycidylated and/or biotinylated dendrimers. Wild-type nematode culture were maintened at 20 °C on NGM agar plates with Escherichia coli OP50 strain as a food source. Nematodes were synchronized by bleaching using hypochlorite. The obtained eggs were left in M9 buffer at 21 °C to hatch until the next day. Then, L1 larvae were placed on NGM agar plates with *E. coli* OP50 strain at 21 °C until they reached the L4 stadium (approximately 44 h). Afterward, the L4 larvae were transferred to 15 mL falcons by washing NGM plates twice with 5 mL water and centrifuged at 1500 rpm for 4 min. After supernatant aspiration, the pellet was re-suspended with 5 mL complete S medium [85] and centrifuged. The supernatant was aspirated again. Then, the density of the nematode suspension was assessed according to Scanlan et al. [86]. Worms were suspended in complete S medium with *E. coli* OP50 (1:1000), 0.08% cholesterol (5 mg/mL in Et-OH), 1% penicillin–streptomycin, 1% nystatin, and 100 mM FUdR (at final concentration 200 µM) to obtain about 20 nematodes in 50 μL. FUdR was added to sterilize the nematodes. After the transfer of nematodes to a 96-well plate (about 20 individuals in 50 μL), the solutions of the studied conjugates in a complete S medium were added (50 μL/well). The plate was incubated at 21 °C for seven days. During this time, live and dead worms were counted under the inverted microscope (Delta Optical IB-100).

### 3.12. Penetration into the Rat Central Nervous System

In this study, 5 male Wistar rats were used. The animals came from the colony of the Experimental Neuropathology Laboratory, Institute of Zoology and Biomedical Research, Jagiellonian University, Krakow, Poland. All animal use procedures were approved by the II Local Ethics Committee for Animal Experiments in Krakow (agreement no. 121/2020) and were performed in accordance with international standards. The animals were bred at a constant temperature (20–21 °C), with unrestricted access to food (standard laboratory diet Labofeed H), and maintained in 12 h/12 h light/dark cycles. On the 60th day, the rats were injected three times every three hours through the tail vein with 1 mL of G4, G4B, G4gl, or G4Bgl solution with a dose of 16 nM/g.b.w. The control group received the same volume of physiological saline solution. After the last administration, the rats were sacrificed by a lethal dose of pentobarbital and perfused transcardially with 0.9% NaCl followed by 10% formalin in 0.1 M phosphate buffer, pH 7.4 (424321737 Chempur). The brains were removed, postfixed for several days, and sectioned into 40 μm thick coronal slices on a vibratome (Leica VT1000S). Then, the slices were covered by mounting medium with DAPI (F6057 Sigma).

### 3.13. Statistical Analysis

To estimate the differences between treated and non-treated control samples, statistical analysis was performed using the non-parametric Kruskal–Wallis test due to the lack of a normal distribution of data in the studied groups (analysis with Shapiro–Wilk test). To determine the statistically significant differences between biotinylated and non-biotinylated conjugates, a Mann–Whitney U test was applied (*p* ≤ 0.05 was considered statistically significant).

To analyze differences in nematode viability between the control, the non-treated group, and the nematodes incubated with conjugates, the Kaplan–Meier estimator was used. Statistically significant differences between the control and treated groups were determined with Gehan’s Wilcoxon test. *p* < 0.05 was considered statistically significant. All analyses and calculations were performed using Statistica 13.3 software (StatSoft, Tulsa, OK, USA).

## 4. Conclusions

Biotinylated and glycidylated PAMAM G4 dendrimers may be efficient drug carriers useful in the therapy of TMZ-resistant glioma indicating the expression of SMVT. G4Bgl showed high biocompatibility in vitro and in vivo. Moreover, it accumulated in glioma cells at the highest degree, where it was distributed in the entire cell area, including the nuclei. It was not a factor inducing drug resistance in glioma cells as in the case of HepG2 liver cancer cells. There are indications that G4Bgl will also penetrate the blood–brain barrier and act in the CNS area. Therefore, it might be a promising candidate for a carrier of therapeutic agents in glioma therapy.

An interesting observation was made regarding biotinylated PAMAM G4. It was very efficiently transported into the body of the *C. elegans* nematode, especially to the embryo region, and therefore can be considered as an excellent drug carrier in anti-nematode and anthelmintic therapy. Research on this phenomenon should be continued.

## Data Availability

The original contributions presented in the study are included in the article; further inquiries can be directed to the corresponding author.
